# Intrinsic Mitochondrial Membrane Potential and Associated Tumor Phenotype Are Independent of MUC1 Over-Expression

**DOI:** 10.1371/journal.pone.0025207

**Published:** 2011-09-23

**Authors:** Michele A. Houston, Leonard H. Augenlicht, Barbara G. Heerdt

**Affiliations:** Department of Oncology, Albert Einstein Cancer Center, Montefiore Medical Center, Bronx, New York, United States of America; Roswell Park Cancer Institute, United States of America

## Abstract

We have established previously that minor subpopulations of cells with stable differences in their intrinsic mitochondrial membrane potential (Δψm) exist within populations of mammary and colonic carcinoma cells and that these differences in Δψm are linked to tumorigenic phenotypes consistent with increased probability of participating in tumor progression. However, the mechanism(s) involved in generating and maintaining stable differences in intrinsic Δψm and how they are linked to phenotype are unclear. Because the mucin 1 (MUC1) oncoprotein is over-expressed in many cancers, with the cytoplasmic C-terminal fragment (MUC1 C-ter) and its integration into the outer mitochondrial membrane linked to tumorigenic phenotypes similar to those of cells with elevated intrinsic Δψm, we investigated whether endogenous differences in MUC1 levels were linked to stable differences in intrinsic Δψm and/or to the tumor phenotypes associated with the intrinsic Δψm. We report that levels of MUC1 are significantly higher in subpopulations of cells with elevated intrinsic Δψm derived from both mammary and colonic carcinoma cell lines. However, using siRNA we found that down-regulation of MUC1 failed to significantly affect either the intrinsic Δψm or the tumor phenotypes associated with increased intrinsic Δψm. Moreover, whereas pharmacologically mediated disruption of the Δψm was accompanied by attenuation of tumor phenotype, it had no impact on MUC1 levels. Therefore, while MUC1 over-expression is *associated with* subpopulations of cells with elevated intrinsic Δψm, it is *not directly linked to* the generation or maintenance of stable alterations in intrinsic Δψm, or to intrinsic Δψm associated tumor phenotypes. Since the Δψm is the focus of chemotherapeutic strategies, these data have important clinical implications in regard to effectively targeting those cells within a tumor cell population that exhibit stable elevations in intrinsic Δψm and are most likely to contribute to tumor progression.

## Introduction

Heterogeneity is a fundamental property of cellular systems, including solid tumors [1], where diversity likely provides reservoirs of cells which, through evasion of preventative or therapeutic intervention and/or tolerance and rapid response to shifting micro-environmental conditions, can participate in tumor expansion and progression. Our previous work has established that within populations of colonic and mammary carcinoma cells there are minor subpopulations of cells that exhibit stable differences in intrinsic mitochondrial membrane potential (Δψm) which are linked to tumorigenic phenotypes [1], [2]. Although the mechanisms involved in generating and maintaining differences in Δψm are unclear, they may reflect alterations in mitochondrial membranes [3]–[8] (Mariadason & Heerdt, unpublished).

The mucin 1 (MUC1) oncoprotein is a highly O-glycosylated heterodimeric, type-I transmembrane glycoprotein that is over-expressed in many cancers [9], [10]. MUC1 is synthesized as a single polypeptide which is cleaved into an N-terminal extra cellular fragment (MUC1 N-ter) and a C-terminal fragment (MUC1 C-ter), which includes transmembrane and cytoplasmic domains. Glycosylation allows MUC1 N-ter to be transported to the cell surface where it is tethered by dimerization with MUC1 C-ter. The cytoplasmic domain of MUC1 C-ter (MUC1-CD) interacts with diverse signal transducing molecules [9], [11]–[14], and has been linked to transcriptional regulation of various genes [15], [16] including VEGF [15]–[17]. Additionally, MUC1 C-ter is targeted to the mitochondria, where it integrates into the outer mitochondrial membrane [13], [14]. Mitochondrial (mt)-associated MUC1 C-ter has been reported to attenuate dissipation of the Δψm and subsequent apoptosis initiated through the intrinsic (mitochondrial mediated) pathway [13]–[15], [18]–[21].

Because MUC1 is over-expressed by most human carcinomas, where it is associated with tumor progression and poor prognosis [22], we asked whether constitutive levels of MUC1 were linked to stable differences in intrinsic Δψm and, as a consequence, were a component or maker of tumor cell heterogeneity in colonic and/or mammary carcinoma cell populations. Using an siRNA approach, we then investigated the impact of diminished levels of MUC1 on intrinsic Δψm and on Δψm linked tumorigenic phenotypes including cellular sensitivity to butyrate (NaB) mediated apoptosis and constitutive hypoxia-independent VEGF secretion. Finally, we investigated the impact of pharmacological alteration of the Δψm on MUC1 and hypoxia-independent VEGF secretion levels. Our data demonstrate that while elevated constitutive MUC1 is *associated with* subpopulations of cells with elevated intrinsic Δψm, MUC1 is *not directly linked to* the generation or maintenance of stable alterations in intrinsic Δψm, or to intrinsic Δψm associated tumor phenotypes.

Chemotherapeutic strategies that preferentially target cells with relatively higher intrinsic Δψm have been investigated and some clinical responses reported [23]–[33]. Based on the data presented here, exploitation of stable elevation in intrinsic Δψm may be a novel and efficacious way to target cells within tumor populations that are the most likely to contribute to tumor expansion and progression [1], [2].

## Materials and Methods

### Cell Culture

The SW620 and SW480 human colonic carcinoma cell lines, and the MCF7 human mammary carcinoma cell line, were obtained from the American Type Culture Collection. Subcloned cell lines were derived as described [1], [2].

### qRT-PCR

Total RNA was purified using Qiagen RNEasy kit (Qiagen, Valencia, CA). cDNA was synthesized using Superscript III First-Strand Synthesis System for RT-PCR (Invitrogen, Carlsbad, CA). MUC1 expression was measured by RT-PCR using Gene Expression Assay (Applied Biosystems, Carlsbad, CA). Expression was normalized to GAPDH.

### Immunoblotting

Mitochondrial enriched fractions and S-10 Post Mitochondrial Fractions (PMF), containing cytoplasmic proteins and light membranes, were prepared as described [34], [35], size-fractionated on 4–20% acrylamide SDS-PAGE gels (Bio-Rad, Hercules, CA) and blotted onto PVDF membranes (Amersham, Arlington Heights, IL). Blots were incubated with anti-MUC1 C-ter (Labvision Ab-5 (MH1; CT2)) and anti-porin (VDAC; Calbiochem; Ab-5) followed by appropriate secondary antibodies. Reactions were detected by Enhanced Chemiluminescence Plus reagents (Amersham) and quantified by densitometry using Kodak IS4000R and Kodak Molecular Imaging Software.

### Transfection

Cells were seeded at 5×10^5^ cells/well in 6 well plates and allowed to grow overnight. The following day cells were either left untreated, mock transfected, transfected with ON-TARGET Plus non-targeting (NT) siRNA pool (Dharmacon, Lafayette, CO), or MUC1 ON-TARGET Plus Smart Pool siRNA (Dharmacon, Lafayette, CO). Transfections were performed using Lipofectamine RNAiMAX (Invitrogen, Carlsbad, CA) and Opti-MEM reduced serum medium (Invitrogen, Carlsbad, CA).

### Quantitation of cell surface MUC1 N-ter

Cells were stained with FITC-conjugated anti-human MUC1 (CD227) monoclonal antibody (BD Pharmingen) and the percentage of stained cells and staining intensity were quantified by flow cytometry.

### Quantitation of Δψm and apoptosis

Mitochondrial membrane potential was determined by flow cytometry using the Δψm-dependent fluorescent dye JC-1 (5,5′6,6′-tetrachloro-1,1′,3,3′ tetraethylbenzimidazol carbocyanineiodide; Molecular Probes, Eugene, OR), analyzed as we have previously described [1], [35]–[38]. Apoptosis was determined by propidium iodide (PI) staining and flow cytometry, as we have described [35], [38], [39].

### Quantitation of vascular endothelial growth factor (VEGF)

Conditioned tissue culture medium was harvested for quantitation of VEGF_165_ protein by ELISA (R&D Systems, Inc., Minneapolis, MN) according to the manufacturer's protocol. The reduction of MTT to formazan [40], combined with a standard curve generated by serial dilution of SW620 cells, was used to determine the number of cells/well and standardize for variations in cell number among wells [41].

### Pharmacologic modulation of Δψm

We have previously shown that exposure of SW620 cells to the K^+^ ionophore valinomycin results in a sustained disruption the Δψm [35], [38]. Thus, following overnight treatment with 5 µM valinomycin, cells were stained with JC-1 or FITC-conjugated anti-human MUC1 (CD227) and analyzed by flow cytometry for quantitation of Δψm and cell surface MUC1 N-ter, respectively. Levels of MUC1 C-ter were determined in mitochondrial enriched fractions by immunoblotting and levels of VEGF_165_ were quantified in harvested cconditioned tissue culture medium (as described above).

### Statistical analyses

Data from at least 3 independent determinations were compared by Bonferroni's multiple comparison tests. Mean data were also evaluated as a function of the intrinsic Δψm, determined by JC-1 staining, using linear regression analyses [1], [36], [41].

## Results

### Constitutive MUC1 levels are associated with stable differences in intrinsic Δψm of subcloned cell lines derived from colonic and mammary carcinoma cell populations

MUC1 over-expression is associated with tumor progression and poor prognosis [22] and is reported to diminish apoptosis signalled through the intrinsic pathway [13]–[15], [18]–[20] and enhance VEGF expression in some cancer cells *in vitro* [17], [42], [43] – cellular activities that we have linked to differences in intrinsic Δψm of subcloned mammary and colonic carcinoma cell lines [1], [2], [41]. Therefore, we investigated the potential relationship between MUC1 levels and intrinsic Δψm.

**Figure 1:**
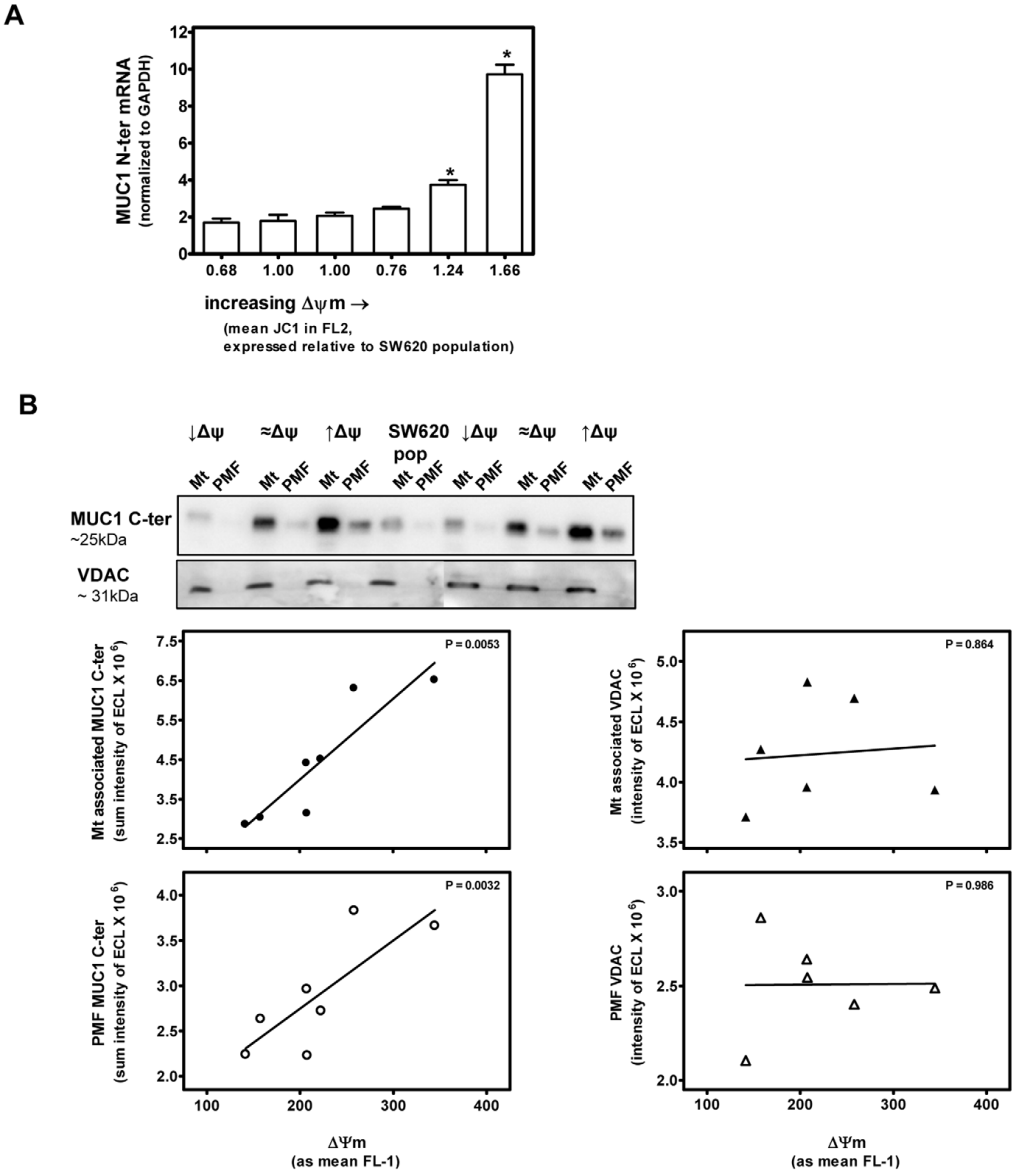
Constitutive MUC1 levels are associated with stable differences in intrinsic Δψm of subcloned cell lines derived from SW620 colonic carcinoma cell population. **A:** Steady state mRNA levels of MUC1 were determined by qRT-PCR, normalized to GAPDH, in the parental population of SW620 cells and in subcloned cell lines derived from SW620 cells that have a range stable intrinsic Δψm. (*; *P*<0.001 vs. the population of SW620 cells.) **B:** Immunoblots of mt and PMF from the SW620 colonic carcinoma cell population and from subcloned lines derived from SW620 cells with intrinsic Δψm higher (↑Δψm), lower (↓Δψm) and comparable (≈Δψm) to that of the population were probed with anti-MUC1 C-ter and anti-VDAC and chemiluminescence signals quantified and expressed as a function of the intrinsic Δψm, determined by JC-1 staining, using linear regression analyses. MUC1 C-ter, but not VDAC, levels in both mt enriched and PMF are significantly associated with the intrinsic Δψm (*P* = 0.0053 and 0.0032, respectively).

Steady state mRNA levels of MUC1 were determined by qRT-PCR in the parental population of SW620 human colonic carcinoma cells and in subcloned cell lines derived from SW620 cells that have stable intrinsic Δψm ranging from higher, comparable or lower than that of the population (↑Δψm, ≈Δψm or ↓Δψm, respectively) [1]. As shown in Figure 1A, MUC1 mRNA levels are at least 1.5 fold, and as much as 6 fold, higher in the subcloned cell lines with elevated Δψm (*P<0.001 vs. the population of SW620 cells).

To investigate whether this relationship between MUC1 mRNA and intrinsic Δψm extended to MUC1 C-ter protein levels, mitochondrial (mt) and accompanying Post Mitochondrial (PMF) enriched fractions were isolated from the SW620 colonic carcinoma cell population and from subcloned lines derived from SW620 cells with a range of relative intrinsic Δψm [1]. Immunoblots of mt and PMF were probed with antibodies directed specifically against the C-ter fragment of MUC1 (MUC1 C-ter) or the mt outer membrane protein VDAC, and chemiluminescence signals quantified and expressed as a function of the intrinsic Δψm, determined by staining cells with the Δψm-dependent dye JC-1, using linear regression analyses. As shown in Figure 1B, while MUC1-C-ter levels in both mt enriched and PMF are significantly associated with the intrinsic Δψm (*P* = 0.0053 and 0.0032, respectively), VDAC levels in neither the mt nor the PMF fractions are linked to intrinsic Δψm. This relationship between MUC1 C-ter and intrinsic Δψm of subcloned cells lines derived from SW620 cells was confirmed by analyses of multiple immunoblots (not shown).

**Figure 2:**
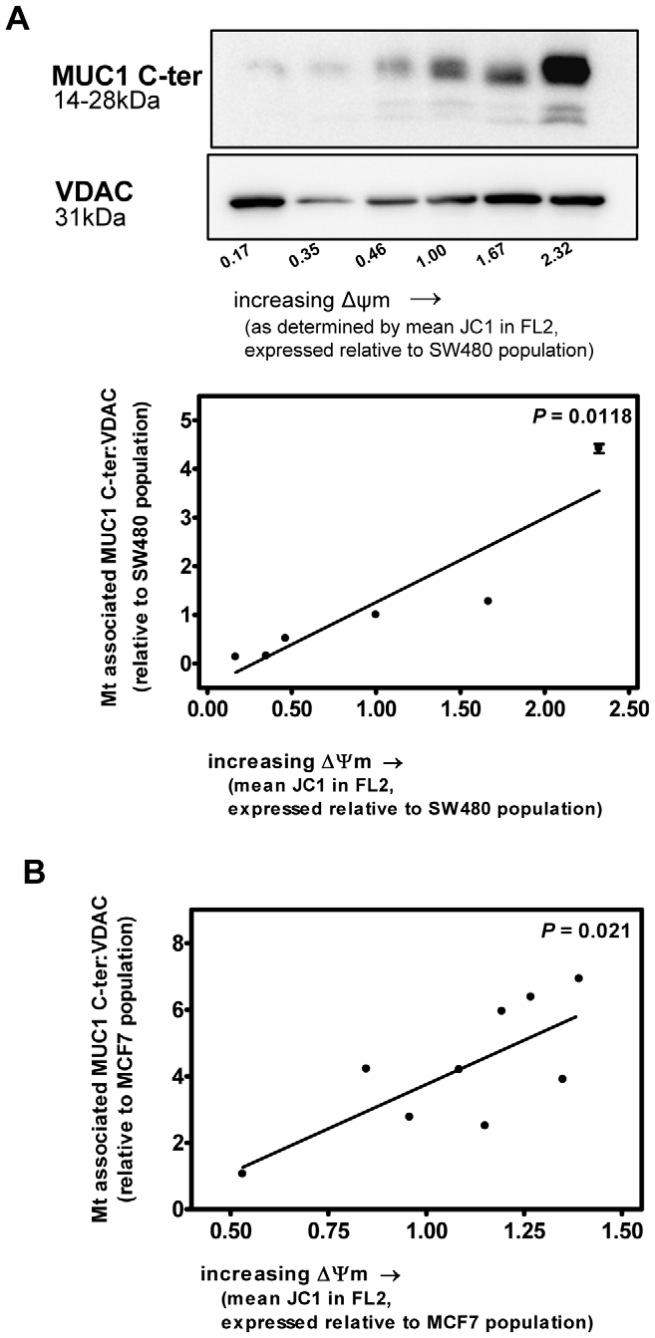
Constitutive mt-associated MUC1 C-ter levels are associated with stable differences in intrinsic Δψm of subcloned cell lines derived from SW480 colonic and MCF7 mammary carcinoma cell populations. Mt-associated MUC1 C-ter levels were determined by immunoblotting in mt enriched fractions isolated from the population of SW480 (**A**) and MCF7 (**B**) cells and from subclones derived from these cell populations that have stable intrinsic Δψm ranging from significantly lower to significantly higher than that of the population. MUC1 C-ter levels are expressed as a function of the relative intrinsic Δψm by linear regression analyses.

We next asked whether the association between intrinsic Δψm and levels of endogenous mt-associated MUC1 C-ter were unique to subclones derived from SW620 colonic carcinoma cells. Thus, we investigated subcloned cell lines with stable differences in intrinsic Δψm that were derived from the SW480 and MCF7 human colonic and mammary carcinoma cell lines, respectively. Similar to the subclones derived from SW620 cells, the SW480 and MCF7 derived subcloned cell lines exhibit intrinsic Δψm-linked tumorigenic phenotypes consistent with enhanced probability to participate in local tumor expansion [2]. Mitochondrial enriched fractions were isolated from the parental populations of SW480 and MCF7 cells and from accompanying derived subcloned cell lines with intrinsic Δψm ranging from significantly lower to significantly higher than that of the population. Mt-associated MUC1 C-ter levels were then determined by immunoblotting and expressed as a function of the relative intrinsic Δψm using linear regression analyses. As shown in Figure 2, constitutive levels of mt-associated MUC1 C-ter are significantly linked to the intrinsic Δψm of subclones established from SW480 colonic, and MCF7 mammary, carcinoma cells (2A and 2B;; *P* = 0.0118 and 0.021, respectively), thereby supporting the relationship between the Δψm and mt associated MUC1 C-ter in cancer cells.

### siRNA mediated down-regulation of MUC1 does not affect intrinsic Δψm

**Figure 3:**
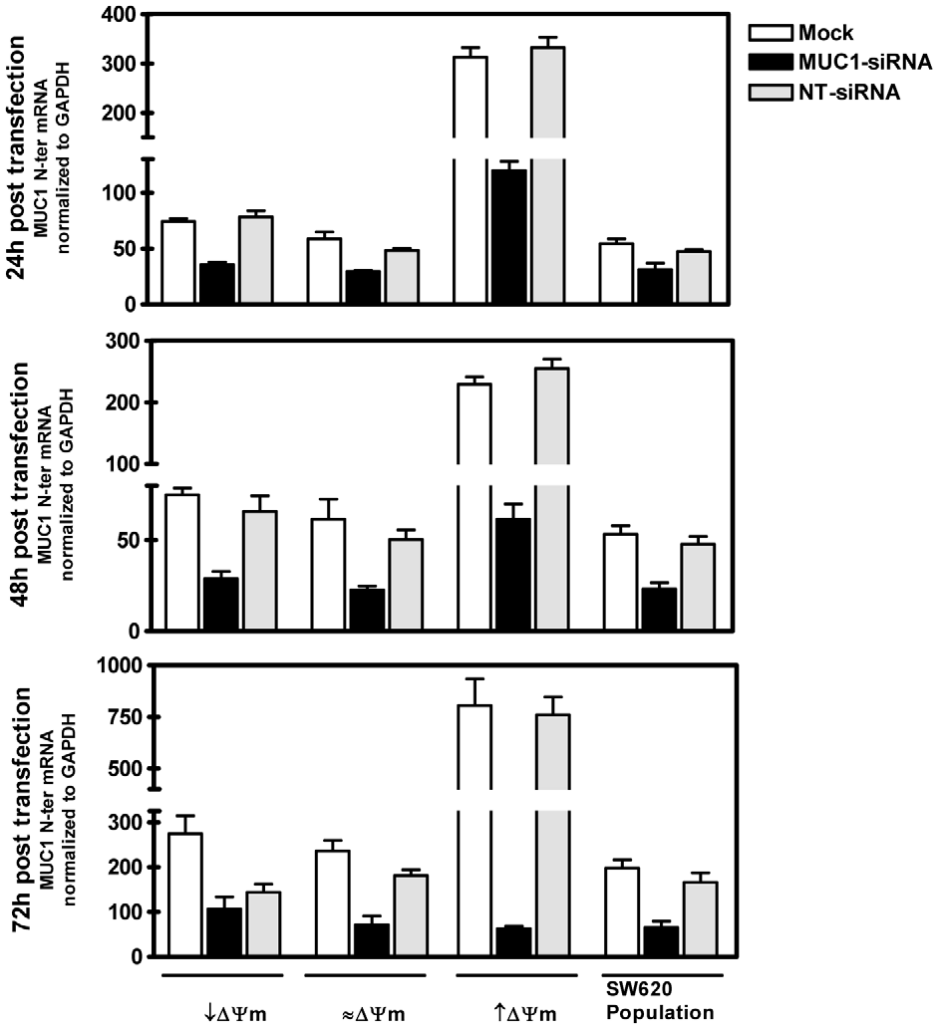
MUC1 siRNA-mediates down regulation of MUC1 expression in subcloned cell lines with different intrinsic Δψm. Subcloned cell lines with intrinsic Δψm higher (↑Δψm), lower (↓Δψm) and comparable (≈Δψm) to that of the population, and the SW620 parental population, were mock transfected, or transfected with NT- or MUC1-siRNA. Twenty-four, 48 and 72 hours later levels of MUC1 mRNA were determined by qRT-PCR, normalized to GAPDH. The level of MUC1 mRNA in the siRNA-MUC1 transfected cell line with ↑Δψm did not differ significantly from that of mock transfected SW620 cell population 48 and 72 hours after transfection (*P*>0.05).

Although the levels of mt-associated MUC1 C-ter are linked to the intrinsic Δψm of cancer cells and MUC1 C-ter has been reported to integrate into the outer mitochondrial membrane [13], [14], it is unclear whether mitochondrial MUC1 C-ter contributes to regulation or maintenance of the Δψm [21] . To investigate this, the SW620 cell population and derived subcloned cell lines with a range of relative intrinsic Δψms (↓Δψm, ≈Δψm and ↑Δψm) were mock transfected, or transfected with non-targeting (NT) or MUC1 siRNA. Twenty-four, 48 and 72 hours later MUC1 mRNA levels were determined by qRT-PCR (Figure 3). Compared to mock or NT-siRNA transfected cells, MUC1 expression levels were down regulated approximately 40% to 60% 24 hours after transfection of MUC-siRNA. Interestingly, the most extensive decrease in MUC1 expression was seen in the subclone with elevated Δψm, in which the original levels of MUC1 were the highest. siRNA-induced down regulation of MUC1 mRNA was maintained 48 and at 72 hours after transfection and, in fact, continued to decrease in each cell line at each time point by approximately 10%, achieving approximately 87% lower levels of MUC1 mRNA in the subclone with elevated intrinsic Δψm after 72 hours. Importantly, although MUC1 expression was not *completely* silenced, levels in the subclone with elevated intrinsic Δψm were comparable to those of the mock transfected population of SW620 cells 48 and 72 hours following MUC1-siRNA transfection MUC1 expression (*P*>0.05).

**Figure 4:**
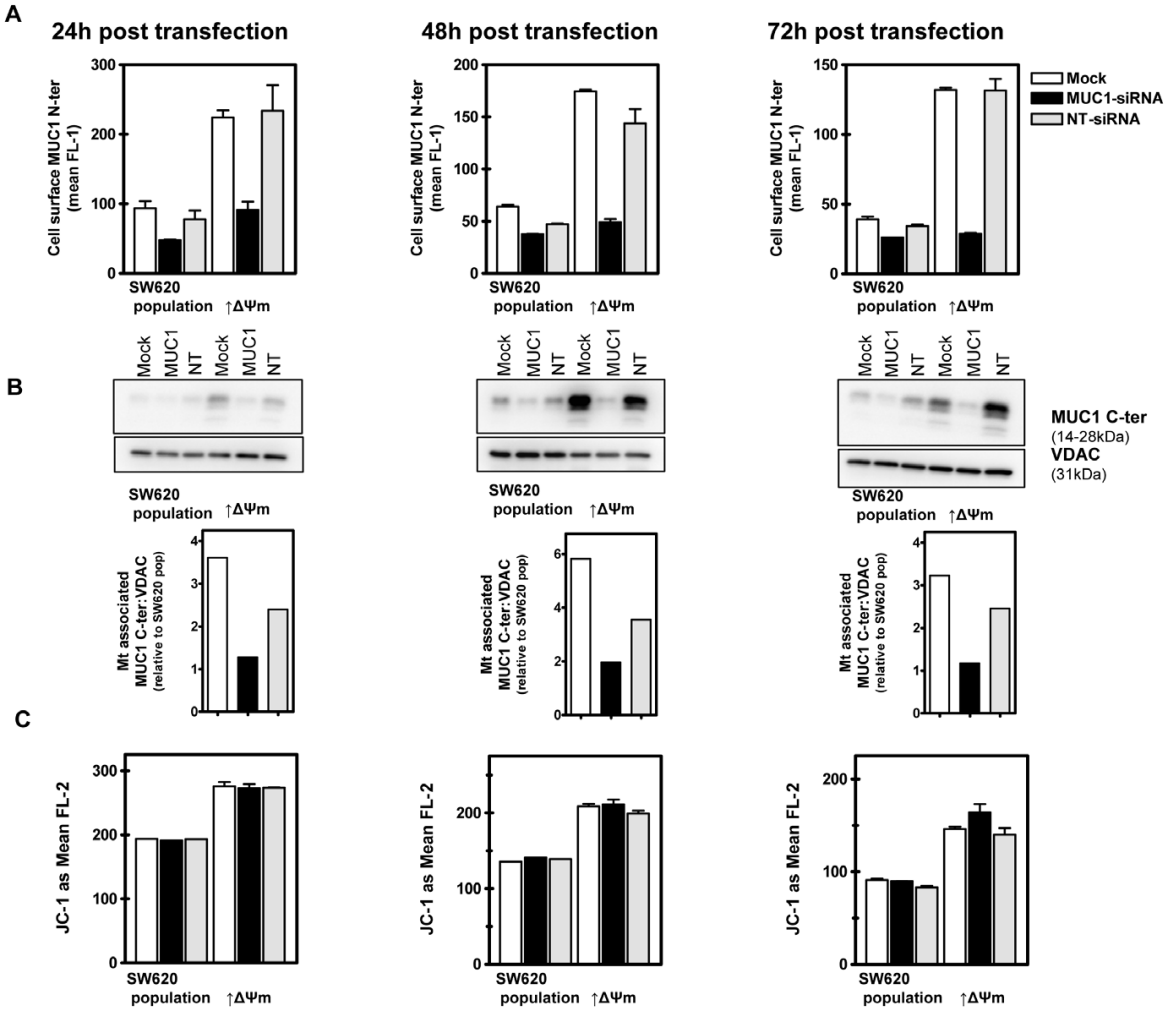
siRNA-mediated down regulation of MUC1 N-ter and mt-associated C-ter levels does not affect the Δψm. Cells were mock, MUC1- or NT-siRNA transfected. Twenty-four, 48 and 72 hours later, levels of cell surface MUC1 N-ter were determined by flow cytometry (**A**); levels of mt-associated MUC1 C-ter determined by quantitative immunoblotting (**B**) and the Δψm was determined by JC-1 staining and flow cytometry.

Because MUC1 mRNA levels in the siRNA-MUC1 transfected subclone with elevated Δψm approximated those of the mock transfected population of SW620 cells, we focused on these two cell lines in subsequent investigations. Thus, cells were mock, NT-siRNA or MUC1-siRNA transfected and 24, 48 and 72 hours later relative levels of cell surface MUC1 N-ter and mt-associated MUC1 C-ter were determined by flow cytometry and immunoblotting, respectively. As shown in Figure 4, siRNA-mediated down regulation of MUC1 mRNA resulted in coincident decreases in cell surface MUC1 N-ter and mt associated MUC1 C-ter in both cell lines at each time point following transfection (4A and 4B, respectively). Moreover, although not *completely* eliminated, consistent with the siRNA induced reduction of MUC1 mRNA (Figure 3), the levels of MUC1 N-ter surface staining and mt-associated MUC1 C-ter in MUC1-siRNA transfected cells with elevated intrinsic Δψm were comparable to those of mock transfected population of SW620 cells at each time point (*P*>0.05). Therefore, siRNA-induced down regulation of MUC1 mRNA is reflected in coincidently diminished levels of cell surface MUC1 N-ter and mt-associated MUC1 C-ter, especially in cells with relatively high intrinsic Δψm.

We next investigated how siRNA mediated down regulation of MUC1 affected the Δψm. Twenty-four, 48 and 72 hours following mock, NT-siRNA or MUC1-siRNA transfection, cells were stained with JC-1 and analyzed by flow cytometry [1], [35]–[38]. As shown in figure 4C, siRNA induced reduction of MUC1 had no effect on the Δψm of either cell line at any time points evaluated. Therefore, despite comparable levels of MUC1 mRNA, cell surface MUC1 N-ter and mt-associated MUC1 C-ter in the subclone with elevated intrinsic Δψm and mock transfected population of SW620 cells – particularly 48 and 72 hours after MUC1-siRNA transfection-the relatively higher intrinsic Δψm was maintained. Similarly, siRNA mediated down regulation had no impact on the Δψm of additional SW620 derived subcloned cell lines with intrinsic Δψm ranging from lower to higher than that of the population (not shown). Thus, it is unlikely that MUC1 levels play a role in the regulation or maintenance of stable difference in intrinsic Δψm.

### siRNA mediated down-regulation of MUC1 does not affect intrinsic Δψm-linked sensitivity to butyrate (NaB) induced apoptosis

Over-expression of MUC1 has been reported to attenuate dissipation of the Δψm and subsequent apoptosis induced through the intrinsic (mitochondrial-mediated) pathway [13]–[15], [18]–[21]. Our previous work [1], [36], [38] has shown that the Δψm, and the process of Δψm dissipation, play critical roles in initiation of the intrinsic apoptotic pathway induced by the physiological relevant [44], chemoprotective [45]–[48] short chain fatty acid butyrate (NaB). Furthermore, we have shown that subpopulations of cells with elevated intrinsic Δψm, derived from either colonic or mammary cancer cell populations, are less sensitive to NaB mediated cytotoxicity than cells with lower Δψm [1], [2], [41].

**Figure 5:**
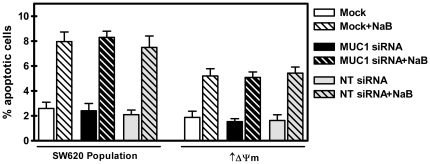
siRNA mediated down-regulation of MUC1 does not affect intrinsic Δψm-linked sensitivity to butyrate (NaB) induced apoptosis. Cells were mock, MUC1- or NT-siRNA transfected and 48 hours later and then exposed to 5 mM NaB for 24 hours. Percentage of apoptotic cells was determined by PI staining and flow cytometry.

To investigate the impact of siRNA mediated down regulation of MUC1 on NaB induced apoptosis, the population of SW620 cells and derived subclone with elevated intrinsic Δψm were mock, NT-siRNA or MUC1-siRNA transfected. Forty-eight hours later, a time point at which the MUC1 levels in two cell lines are comparable (Figures 3 and 4A and 4B), cells were exposed to 5mM NaB, a physiologically relevant concentration [49], [50], for 48 hours, a time point at which we have shown induction of significant apoptosis [1], [36], [38]. Cells were then stained with PI and the percentage of apoptotic cells determined by flow cytometry. As shown in Figure 5, down regulation of MUC1 did not significantly impact NaB mediated apoptosis in either cell line, and, consistent with the absence of an effect on intrinsic Δψm (Figure 4C), decreased sensitivity to NaB was retained in MUC1-siRNA transfected cells with elevated intrinsic Δψm.

### siRNA mediated down-regulation of MUC1 does not affect intrinsic Δψm-linked constitutive hypoxia-independent VEGF secretion

**Figure 6:**
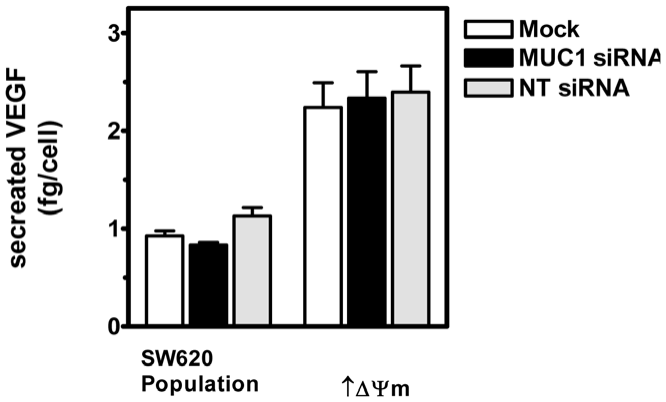
siRNA mediated down-regulation of MUC1 does not affect intrinsic Δψm-linked constitutive hypoxia-independent VEGF secretion. Cells were mock, MUC1- or NT-siRNA transfected and 48 hours later VEGF secretion levels were determined in harvested conditioned tissue culture medium.

We have previously shown a significant association between the intrinsic Δψm and levels of steady state, hypoxia-independent VEGF secretion in subcloned cell lines derived from mammary and colonic carcinoma cells [1], [2], [41]. Because the cytoplasmic domain of MUC1 C-ter (MUC1-CD) has been linked to transcriptional regulation of VEGF [15]–[17] and our data show that the levels of MUC1 C-ter in the PMF – which encompasses cytoplasmic components - are related to the intrinsic Δψm (Figure 1B), we investigated the effect of siRNA induced MUC1 down regulation on steady state VEGF secretion in colonic carcinoma cells with different Δψm. Thus, 48 hours after siRNA transfection, a time point at which the MUC1 levels in cells with elevated Δψm are comparable to those in mock transfected cells with lower intrinsic Δψm (Figures 3 and 4A and 4B), conditioned tissue culture medium was harvested and levels of VEGF were determined. Similar to the absence of effects on intrinsic Δψm (Figure 4C) or NaB induced apoptosis (Figure 5) down regulation of MUC1 did not significantly affect constitutive hypoxia-independent VEGF secretion levels in either cell line, with the relatively higher level of secretion retained in MUC1-siRNA transfected cells with elevated intrinsic Δψm (Figure 6).

### Pharmacologically mediated disruption of the Δψm does not affect MUC1 levels but attenuates intrinsic Δψm-linked constitutive hypoxia-independent VEGF secretion

**Figure 7:**
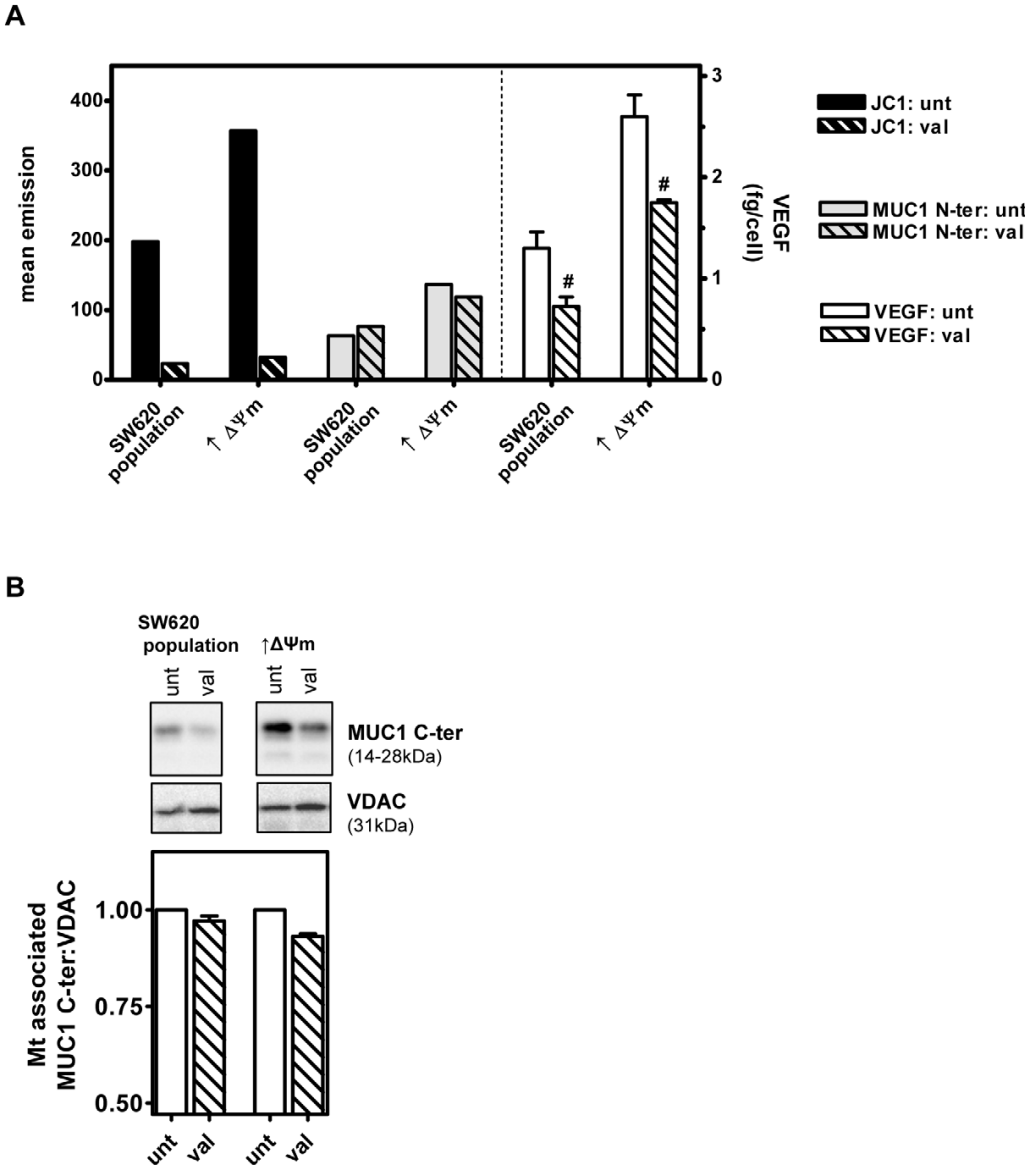
Pharmacologically mediated disruption of the Δψm does not affect MUC1 levels but attenuates intrinsic Δψm-linked constitutive hypoxia-independent VEGF secretion. Cells were exposed to 5 µM valinomycin overnight and then stained with JC-1 or FITC-conjugated anti-human MUC1 (CD227) and analyzed by flow cytometry for quantitation of Δψm and cell surface MUC1 N-ter, respectively. Levels of VEGF_165_ were quantified in conditioned tissue culture medium harvested from untreated and valinomycin treated cells (#; *P*<0.05 valinomycin vs. untreated) (**A**). Levels of MUC1 C-ter were determined in mitochondrial enriched fractions by immunoblotting (**B**).

Finally, to investigate the impact of disruption of the intrinsic Δψm on MUC1 and constitutive hypoxia-independent VEGF secretion levels, the population of SW620 cells and derived subclone with elevated intrinsic Δψm were exposed to the K^+^ ionophore valinomycin to dissipate the Δψm [35], [38]. The Δψm, secreted VEGF, MUC1 N-ter and MUC1 C-ter levels were then determined. As shown in Figure 7A, whereas valinomycin treatment resulted in decreases in the Δψm of both cell lines by at least 90%, accompanied by significantly decreased constitutive VEGF secretion levels (#, *P*<0.05), neither cell surface MUC1 N-ter nor mt-associated MUC1- C-ter (Figure 7B) levels were impacted in either cell line by disruption of the Δψm. Thus, it is likely that the intrinsic Δψm and associated tumor phenotype are independent of MUC1 over-expression in colonic carcinoma cells.

## Discussion

Heterogeneity among cells is a fundamental property of tumors and likely allows for selection of subpopulation phenotypes that have an increased probability of participating in subsequent tumor progression [51]. Whereas the origins of, and the mechanisms by which, cellular heterogeneity contributes unequally to tumorigenesis are unclear [51], it has been know for some time that the *population average* Δψm of cancer cells is significantly higher than that of normal cells ([24], [52]–[55] and that mitochondria are important and critical elements in establishing tumorigenic phenotype [56].

Using single cell subclones, our previous work has established the existence of subpopulations of cells within mammary and colonic carcinoma cell populations that, compared to the population average Δψm, have stable elevations in their intrinsic Δψm [1], [2]. Moreover, we have shown that these elevations in intrinsic Δψm are linked to tumor phenotypes associated with increased probability of tumor progression [1], [2]. Because MUC1 is over-expressed in many human carcinomas and cell lines where it is also associated with tumor progression [9], [22], we investigated the relationship between intrinsic Δψm and endogenous MUC1 expression. We found that constitutive MUC1 levels are significantly associated with stable differences in intrinsic Δψm of subcloned cell lines derived from colonic and mammary carcinoma cell populations, with higher levels of MUC1 exhibited in cells with elevated intrinsic Δψm. However, we also found that siRNA-induced down-regulation of MUC1 in colonic carcinoma cells with elevated intrinsic Δψm to levels comparable to those in cells with lower Δψm does not affect the intrinsic Δψm or Δψm-linked tumor phenotypes, including the decreased sensitivity to chemoprotective NaB induced apoptosis and the increased constitutive, hypoxia-independent VEGF secretion that are characteristic of cells with elevated intrinsic Δψm [1], [2]. Additionally, we found that chemically induced disruption of the Δψm was associated with decreased levels of constitutive hypoxia-independent VEGF secretion, but had no impact on MUC1 levels. Therefore, these data suggest that over-expression of MUC1 is not directly involved in the generation or maintenance of stable alterations in intrinsic Δψm and Δψm linked phenotypes, and that the intrinsic Δψm and associated tumor phenotype are independent of MUC1 over-expression in colonic carcinoma cells.

The higher average Δψm in cancer cells [24], [52]–[55] has been used as a rationale to develop compounds that accumulate in the mt based on the Δψm (i.e., mitochondriotropic agents) to preferentially kill tumor cells [23]–[33]. Because mitochondriotropic agents accumulate in the mitochondrial matrix at a 10-fold higher level with each 60 mV increase in Δψm [57], they have the potential of being particularly efficacious in targeting cells with modest elevations in the intrinsic Δψm-the specific cells within the tumor cell population that our data show are the most likely to participate in tumor expansion and progression [1], [2].
